# Study of Several Alginate-Based Hydrogels for In Vitro 3D Cell Cultures

**DOI:** 10.3390/gels8030147

**Published:** 2022-02-27

**Authors:** Weijie Jiao, Xiaohong Li, Jingxin Shan, Xiaohong Wang

**Affiliations:** 1 Center of 3D Printing & Organ Manufacturing, School of Intelligent Medicine, China Medical University (CMU), Shenyang 110122, China; 18900920356@163.com (W.J.); bruceli20211129@163.com (X.L.); shanjingxin@huh.edu.cn (J.S.); 2 Department of Biomedical Engineering, HE University, Shenyang 110163, China; 3 Center of Organ Manufacturing, Department of Mechanical Engineering, Tsinghua University, Beijing 100084, China

**Keywords:** pathological model, alginate-based hydrogels, microscopic structure, cytocompatibility, in vitro 3D cell culture

## Abstract

Hydrogel, a special system of polymer solutions, can be obtained through the physical/chemical/enzymic crosslinking of polymer chains in a water-based dispersion medium. Different compositions and crosslinking methods endow hydrogel with diverse physicochemical properties. Those hydrogels with suitable physicochemical properties hold manifold functions in biomedical fields, such as cell transplantation, tissue engineering, organ manufacturing, drug releasing and pathological model analysis. In this study, several alginate-based composite hydrogels, including gelatin/alginate (G-A), gelatin/alginate/agarose (G-A-A), fibrinogen/alginate (F-A), fibrinogen/alginate/agarose (F-A-A) and control alginate (A) and alginate/agarose (A-A), were constructed. We researched the advantages and disadvantages of these hydrogels in terms of their microscopic structure (cell living space), water holding capacity, swelling rate, swelling–erosion ratio, mechanical properties and biocompatibility. Briefly, alginate-based hydrogels can be used for three-dimensional (3D) cell culture alone. However, when mixed with other natural polymers in different proportions, a relatively stable network with a good cytocompatibility, mechanical strength and water holding capacity can be formed. The physical and chemical properties of the hydrogels can be adjusted by changing the composition, proportion and cross-linking methods of the polymers. Conclusively, the G-A-A and F-A-A hydrogels are the best hydrogels for the in vitro 3D cell cultures and pathological model construction.

## 1. Introduction

The normal survival, proliferation, differentials and communication of eucaryote cells are inseparable from the regulation of their surrounding microenvironment, which is mainly composed of body fluids, growth factors and extracellular matrices (ECMs). In order to satisfy the in vitro construction requirements of bioartificial tissues/organs and bio-pathological models, it is vitally important to imitate the constituents (ingredients or components) of the ECMs [1]. Hydrogel, a colloidal gel in which water is the dispersion medium of polymers, can better simulate the surrounding microenvironments of living cells and has wide applications in biomedical fields, such as cell transplantation, tissue engineering, organ manufacturing, drug releasing and pathological analysis [2]. In particular, the physicochemical properties of hydrogels can be easily adjusted through various physical (e.g., heating, light), chemical (e.g., ion catalysis) and biochemical (e.g., enzymatic) crosslinking reactions [3].

**Figure 1:**
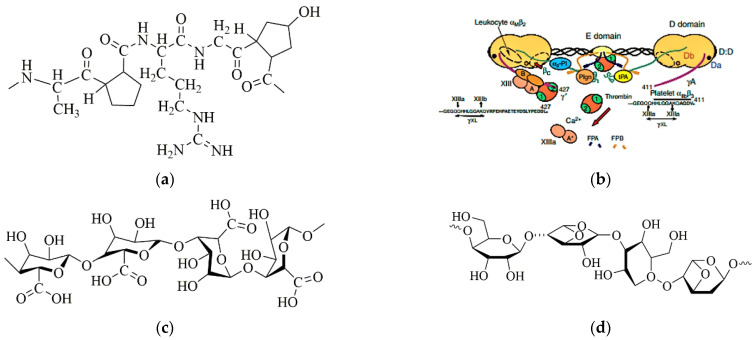
Molecular structures of several nature polymers: (**a**) gelatin; (**b**) fibrinogen; (**c**) alginate; (**d**) agarose.

Gelatin, semi-degraded from collagen with no fixed structure and molecular weight, can be widely found in animal skin, bone, muscle membrane, muscle charm and other connective tissues, and often presents as white or flaxen, translucent, microstrip glossy flake or powder states after purified (Figure 1a). It is a colorless, odorless, involatile, transparent and hard amorphous substance that is soluble in hot water and can absorb water 5–10 times its weight [4,5,6].

Fibrinogen, the precursor of fibrin widely found in most organisms as a key substance for blood coagulation, is a large, complex, fibrous glycoprotein that has three pairs of polypeptides linked together by 29 disulfide bonds (Figure 1b). It has a spherical region at each end which is connected by an alpha helical coil rod in the middle of the polypeptide chain. During the final stage of clotting, soluble fibrinogen is converted to insoluble fibrin, which clots the blood [7,8,9]. As a natural polymer, fibrinogen has an excellent biocompatibility, rapid biodegradability, moderate water retention capability and reliable mechanical property for a wide range of biomedical applications [10,11,12,13].

Alginate, a natural anion polysaccharide derived from the cell wall and intercellular mucilage of brown algae, is a linear polymer consisting of (1→4)-β-linked D-mannuronic acid and (1→4)-α-linked L-guluronic acid, with a relative molecular weight of approximately 10^6^ (Figure 1c). A single alginate molecule tends to have three zones, namely the “M-zone” (rich in mannuronic acid), the “G-zone” (rich in guluronic acid) and the “MG zone” (rich in both types of uronic acids). Ca^2+^ and other bivalent cations are easily bound to the “G-zone”, leading to the chemical crosslinking of alginates with an interpenetrating three-dimensional (3D) network [14,15,16].

Agarose, a linear polymer whose basic structure is a long chain of alternating 1,3-linked β-d-galactose and 1, 4-linked 3, 6-endoether-L-galactose, is hydrophilic and almost completely free of charged groups (Figure 1d). Agarose can dissolve in water when it is heated above 90 °C, and forms a semi-solid hydrogel through hydrogen bonding when the temperature drops to 35–40 °C [17,18,19]. The physicochemical properties of agarose hydrogels are usually expressed in terms of its mechanical strength. The higher the mechanical strength, the better the gel performance. Traditionally, agarose hydrogels are used as bacteria culture media, which seldom cause denaturation of the biological macromolecules.

Each of the above mentioned single polymer hydrogels has unique advantages and disadvantages in mimicking the ECMs [20,21,22]. For example, sodium alginate hydrogel has a high mechanical strength and sufficient elasticity after crosslinking by Ca^2+^. Therefore, it can be used as a good strength supporting material and can 3D culture cells in vitro. Nevertheless, it needs the assistance of other nature polymers to mimic the ECMs, and to achieve an excellent biocompatibility. Fibrinogen and gelatin hydrogels have excellent biocompatibilities and water infiltrabilities, but their mechanical strengths are pretty low. Agarose hydrogel has a high mechanical strength and strong stability, but it has an insufficient biocompatibility for in vitro eucaryote 3D cell cultures.

In our previous studies, we have created a series of 3D printing and combined mold technologies for bioartificial organ manufacturing [23,24,25]. Several natural hydrogels, such as alginate, fibrinogen, chitosan and gelatin, have been used frequently as ECMs for multi-hierarchical vascular/neural construction and homogeneous/heterogeneous cell accommodation. In this study, we have systematically researched the physicochemical properties and biocompatibility of several alginate-based hydrogels, such as alginate (A), alginate/agarose (A-A), gelatin/alginate (G-A), gelatin/alginate/agarose (G-A-A), fibrinogen/alginate (F-A) and fibrinogen/alginate/agarose (F-A-A), for in vitro 3D cell cultures. These selected hydrogels have great potential in their applications toward solving the bottleneck problems that have perplexed tissue engineers, biomaterial researchers, stem cell specialists, pharmaceutists and other scientists for several decades.

## 2. Results

### 2.1. Optimization of the Components and Proportions of the Hydrogels

Four primary components and proportions of the composite hydrogels are shown in Table 1.

A series of preliminary experiments were conducted to optimize the best components and proportions. Based on the water holding capacity, swelling rate, swelling-erosion ratio, mechanical strength, cell proliferation rate and biocompatibility of the samples, several groups from Table 1 with proper polymer components and proportions were selected and shown in Table 2. These hydrogels were used for later in vitro 3D cell cultures.

### 2.2. Scanning Electron Microscope (SEM) Observation

The microstructure of hydrogel determines whether the cells have a suitable living space in the process of the 3D cultures, and whether the hydrogel can accommodate sufficient nutrients to support cell growth and waste discharge. A regular and uniform pore structure is favorable for cells to attach to polymer networks and to form stable and dense tissues.

**Figure 2:**
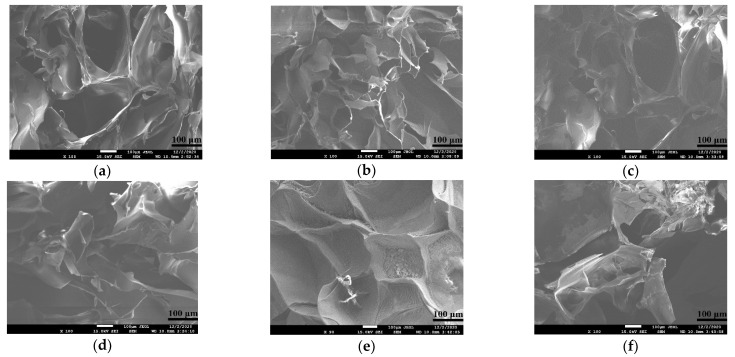
Scanning electron microscope images of the optimal hydrogels prepared in Table 1 after freeze-drying: (**a**) micropore structure of the G-A hydrogel; (**b**) micropore structure of the G-A-A hydrogel; (**c**) micropore structure of the F-A hydrogel; (**d**) micropore structure of the F-A-A hydrogel; (**e**) micropore structure of the A hydrogel; (**f**) micropore structure of the A-A hydrogel.

The microstructures of several alginate-based hydrogels in Table 2 are shown in Figure 2. The microstructures of A and A-A (Figure 2e,f) are messy. Some pores are very large, some are very small and some of them even stick together to form thick hydrogel wall, affecting the entry and exit of nutrients, so they are not suitable to be used as a molding material alone. The addition of gelatin and fibrinogen makes the pore’s morphology of the G-A and F-A (Figure 2a,c) relatively regular, with a large pore scope, large number of pores and uniform wall thickness, suggesting that it can be used as a 3D cell culture material. Meanwhile, the G-A-A and F-A-A structures (Figure 2b,d) obtained by adding agarose are more regular and the pore shape is reduced, but the number of pores is relatively increased and the wall thickness is comparatively uniform, which can provide cells with more attachment sites and larger growth rooms.

### 2.3. Water Holding Capacity (WHC)

**Figure 3:**
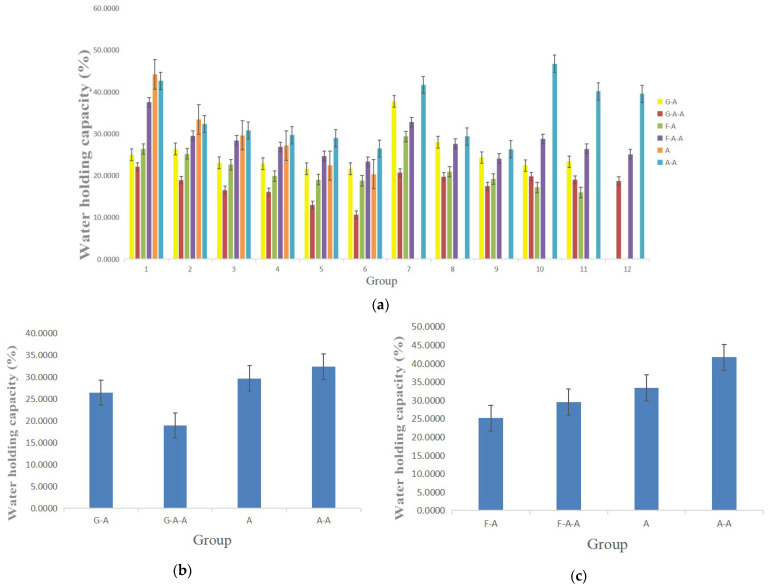
Water holding capacity (*WHC*) of the alginate-based hydrogels: (**a**) comparison of the *WHC*s of the G-A, G-A-A, F-A, F-A-A, A and A-A hydrogels prepared in Table 1; (**b**) comparison of the *WHC*s of the G-A, G-A-A, A and A-A hydrogels prepared in Table 2; (**c**) comparison of the *WHC*s of the F-A, F-A-A, A and A-A hydrogels prepared in Table 2.

The water holding capacity (*WHC*) refers to the amount of water retained per unit of dry matter. It is a significant physiochemical property of hydrogel. The *WHC* of the alginate-based hydrogels in Table 1 is shown in Figure 3. With the increase in each polymer concentration, including alginate itself, all of the *WHC*s of the hydrogels decrease gradually. Both gelatin and fibrinogen lower the *WHC*s of the alginate-based hydrogels, obviously. The effect of fibrinogen on the *WHC*s is significantly higher than that of gelatin. The addition of agarose in the A and F-A hydrogel plays a critical role in improving the *WHC*s of the alginate-based hydrogels. Nevertheless, the addition of agarose in the G-A hydrogel weakens the *WHC*s. Most of the *WHC*s of the hydrogels in Table 1 are between 20–40 times their own dry weight, indicating that the selected hydrogels have enough water molecules for in vitro 3D cell cultures.

### 2.4. Swelling Rate (SR)

The capability to swell after absorbing liquid is another important property of hydrogels used in 3D cell cultures. The (*SR*) can effectively reflect the nutrient and metabolite infiltration and transportation capabilities of the hydrogels. 

**Figure 4:**
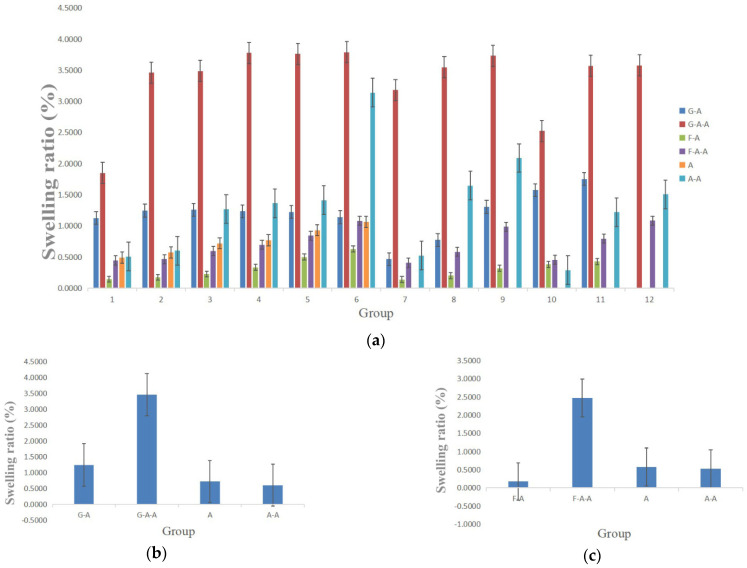
Swelling ratio of the alginate-based hydrogels: (**a**) comparison of the swelling rates of the G-A, F-A, A-A, G-A-A, F-A-A and A hydrogels prepared in Table 1; (**b**) comparison of the swelling rates of the G-A, G-A-A, A and A-A hydrogels prepared in Table 2; (**c**) comparison of the swelling rates of the F-A, F-A-A, A and A-A hydrogels prepared in Table 2.

The *SR*s of the alginate-based hydrogels are shown in Figure 4. With the increase in each component in the alginate-based hydrogels, the *SR*s all demonstrate an increasing trend. Both the gelatin and fibrinogen have distinctively enhanced the swelling rates of the alginate-based hydrogels. The effect of gelatin on the *SR*s was significantly higher than that of fibrinogen. The addition of agarose has also increased the *SR*s of the alginate-based hydrogels. In particular, the *SR*s of the A and A-A hydrogels are between 0–1.0, whereas those of the G-A hydrogels are between 1.0–1.5, those of the G-A-A are between 3.0–3.5, those of the F-A are between 0–0.5 and those of the F-A-A are between 2.0–2.5.

### 2.5. Swelling-Erosion Ratio

Degradability is another vital property of hydrogels. For a 3D cell culture, the swelling-erosion ratio should be adjusted appropriately according to the purpose of use.

**Figure 5:**
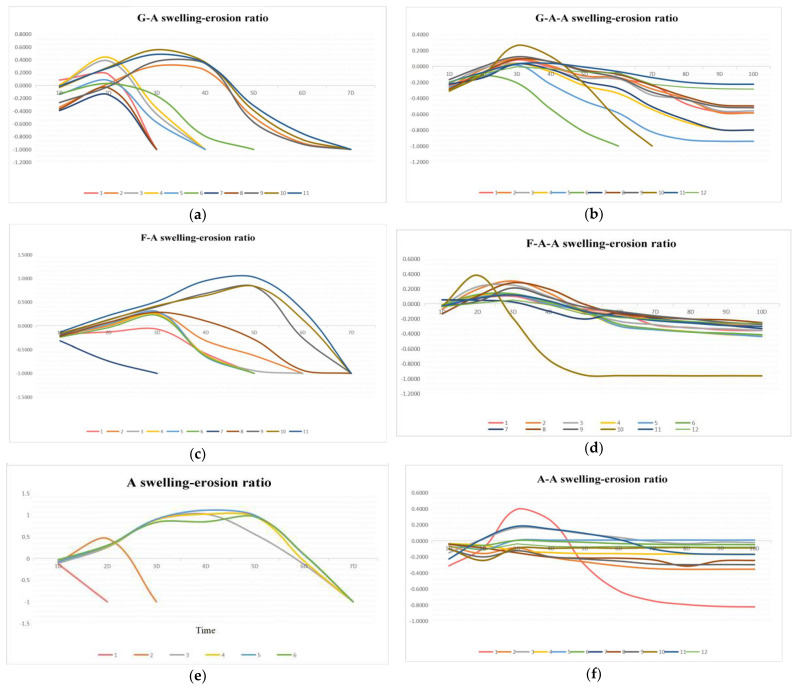
Swelling-erosion ratio of the alginate-based hydrogels: (**a**) G-A hydrogel; (**b**) G-A-A hydrogels; (**c**) F-A hydrogels; (**d**) F-A-A hydrogels; (**e**) A hydrogels; (**f**) A-A hydrogels.

The swelling-erosion ratio of the alginate-based hydrogels within several days are shown in Figure 5. The increase of the values in the curves represents the swelling, while the decrease represents the erosion. With the increase in the alginate concentration, the swelling-erosion ratio of the hydrogel is effectively delayed. Alginate hydrogels can be completely degraded within 7 days (Figure 5e). The addition of gelatin and fibrinogen can accelerate the swelling–erosion ratio of the hydrogels (Figure 5a,c), whereas the addition of agarose can significantly slow down the swelling-erosion ratio of the hydrogels (Figure 5b,d,f). When the agarose concentration reaches 0.5% (*w*/*v*), the swelling–erosion ratio can be guaranteed within ten days. The higher the agarose concentration, the smoother the degradation curves. When the concentration of agarose reaches a certain value, the hydrogels do not degrade for a long time (i.e., more than 12 days) (Figure 5f). However, when the concentration of agarose is very low, its influence on the swelling–erosion ratio is slight and can be neglected.

### 2.6. Mechanical Properties

Hydrogels with different hardnesses can be used for different purposes. For example, most of the hydrogels can be used for soft tissue/organ construction because their hardnesses are similar. For tumor model construction (or modeling) harder hydrogels should be selected for malignant tumors because their hardnesses are higher than those of benign tumors. Therefore, different combinations and proportions of hydrogels can be chosen and optimized according to the characteristics of the tissues/organs or pathological models to be constructed.

**Figure 6:**
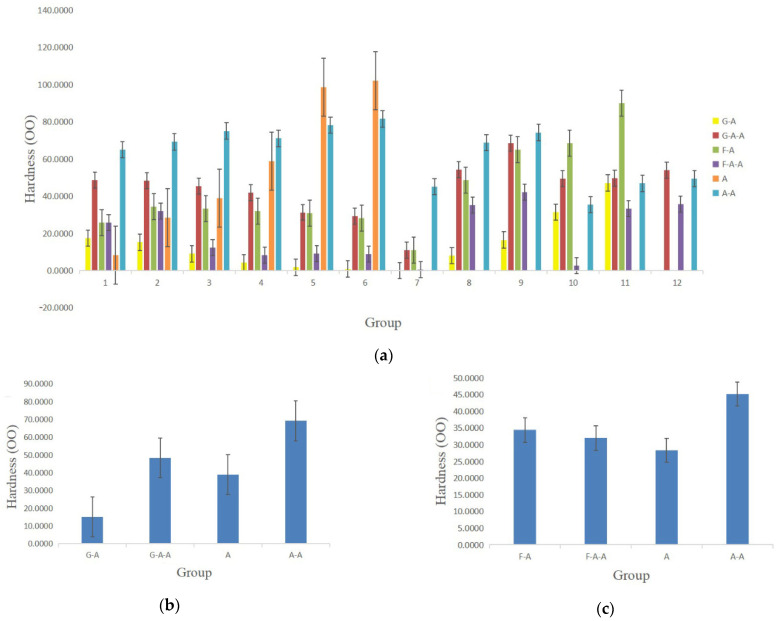
Hardness of the alginate-based hydrogels: (**a**) alginate (A), alginate/agarose (A-A), gelatin/alginate (G-A), gelatin/alginate/agarose (G-A-A), fibrinogen/alginate (F-A) and fibrinogen/alginate/agarose (F-A-A) groups with different components and ratios; (**b**) G-A, G-A-A, A and A-A hydrogels in Table 2; (**c**) F-A, F-A-A, A and A-A hydrogels in Table 2.

The hardness of the alginate-based hydrogels prepared in Table 1 is shown in Figure 6. With the increase in the gelatin and fibrinogen concentrations, the hardness of the alginate-based hydrogels decreases continuously (Figure 6a). The influence of gelatin on the hardness is obviously higher than that of fibrinogen. Meanwhile, the hardness of the hydrogels increases with the increase in the alginate and agarose concentrations. The hardness of the G-A hydrogel with the optimal concentration, as shown in Table 2, is approximately 15 OO, whereas the respective value is approximately 50 OO for G-A-A, 35 OO for F-A and 30 OO for F-A-A (Figure 6b,c).

### 2.7. In Vitro 2D Cell Cultures

**Figure 7:**
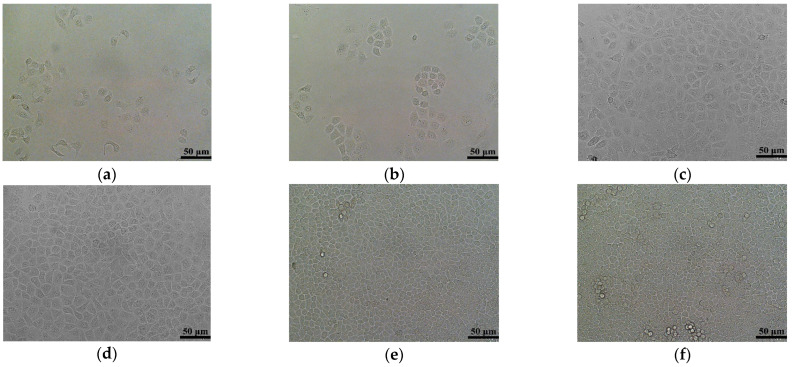
(**a**) A two-dimensional (2D) cell culture image on day 1. (**b**) A 2D cell culture image on day 3. (**c**) A 2D cell culture image on day 5. (**d**) A 2D cell culture image on day 7. (**e**) A 2D cell culture image on day 10. (**f**) A 2D cell culture image on day 13.

Cell states in 2D planar cultures are shown in Figure 7. As the cell culture time increases, the cell number increases greatly. The cell density on day 3 is nearly twice that on day 1. The culture flask is completely covered by the cells on day 7. Cells on the bottom of the culture flask squeeze each other on day 10 to occupy the living space. There are some cells detached from the bottom of the culture flask. On day 13, there are a large amount of darker and brighter cell clumps detached from the bottom of the culture flask. This phenomenon indicates that the dead cell clumps float in the medium. At this time, the cell density does not increase but a large amount of cells die.

### 2.8. Cell Proliferation Rate in the Hydrogels

The cell proliferation rate reflects the proliferation capability of cells in the hydrogel. A suitable hydrogel material must satisfy the cells in many aspects, so that it can be used for tissue/organ formation and tumor model construction. In order to visually observe the advantages of the 3D cultures relative to the flat culture, the proliferation rate of cells was tested using CCK-8 Cell Counting Kit, and the test results were obtained.

**Figure 8:**
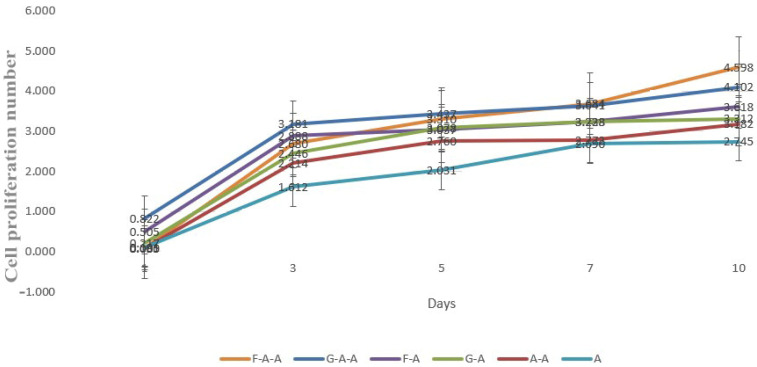
Cell proliferation states in different hydrogels.

From Figure 8, it can be noticed that the cells in the hydrogels in each group have been in a state of continuous proliferation, and the cells proliferated rapidly in one to three days. Compared with day one, the proliferation number on day ten in the F-A-A group, G-A-A group, F-A group and G-A group was 4.6 times, 4.1 times, 3.6 times, 3.3 times, respectively. The A group was 3.1 times, whereas the A-A group was 2.2 times.

### 2.9. Cell States in the Hydrogels

Acridine orange (AO)/propidium iodide (PI) staining is used to react with cells. When cells in hydrogels are alive, they are observed as green dots under a laser confocal microscoope (LSM). Meanwhile, when the cells are dead, they are detected as red dots.

**Figure 9:**
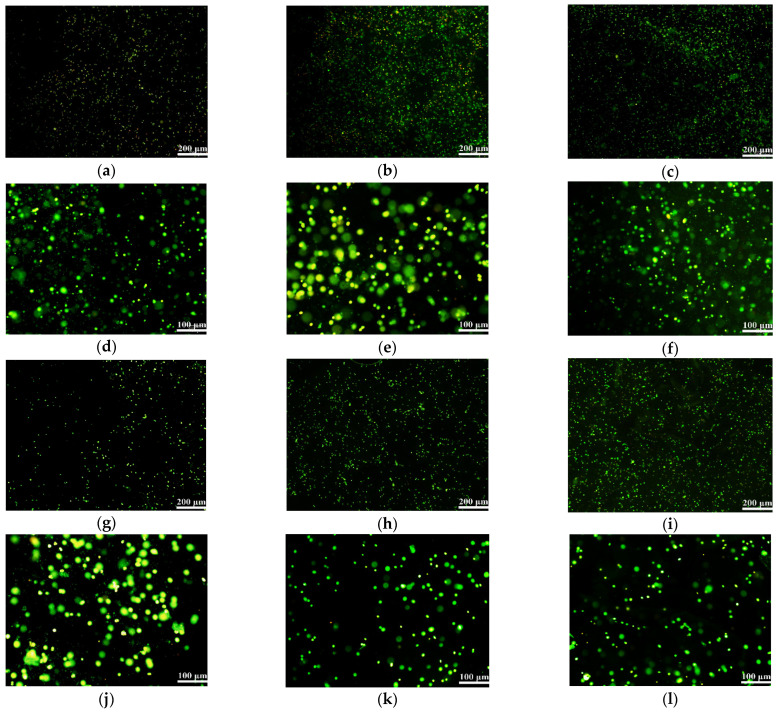
Laser confocal microscoope (LSM) images of AO/PI stained TCA-8113 cells embedded in hydrogels after 1 day: (**a**) G-A hydrogel: the cells are all alive and stained in green; (**b**) G-A-A hydrogel: most cells are green, with some in bright yellow; (**c**) F-A hydrogel: cells are alive and stained in green; (**d**) enlarged image of (**a**); (**e**) enlarged image of (**b**); (**f**) enlarged image of (**c**); (**g**) F-A-A hydrogel: most cells are alive and stained in green; (**h**) A hydrogel: most cells are alive and stained in green; (**i**) A-A hydrogel: cells are alive in green; (**j**) enlarged image of (**g**); (**k**) enlarged image of (**h**); (**l**) enlarged image of (**i**).

Figure 9 shows that after cells were embedded in the hydrogels and cultured for one day, all of the green blips were evenly distributed in the field of vision, and red blips were rarely seen. This means that the cells are basically all alive and are almost uniformly distributed in the hydrogels. The black part represents the ECM mimicking alginate-based hydrogels. By zooming in on the image, it can be clearly seen that the cells grow and proliferate well in the 3D hydrogels. In particular, different layers of the cells can be clearly seen under the microscope, indicating that all of the cells, even deep inside the hydrogels, can survive well inside the hydrogels at this time point.

**Figure 10:**
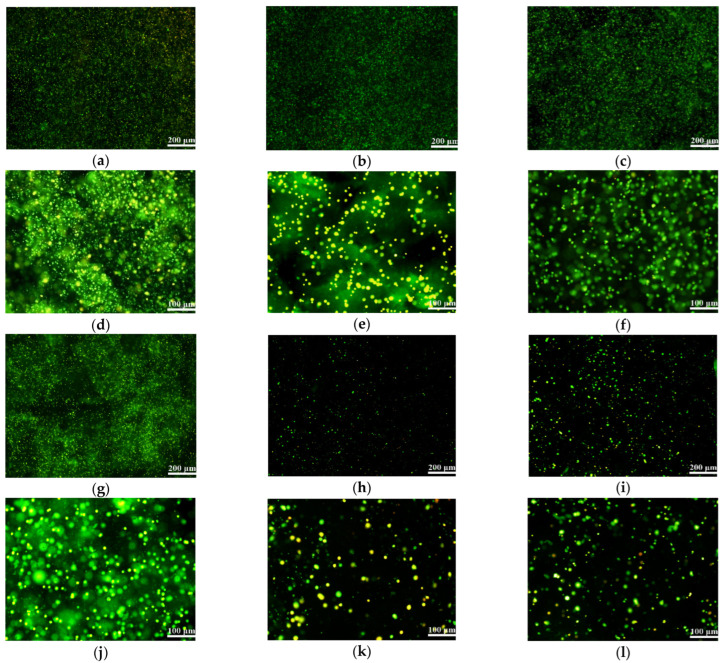
Laser confocal microscoope (LSM) images of AO/PI stained TCA-8113 cells embedded in hydrogels after 10 days: (**a**) G-A hydrogel: nearly all of the cells are all alive and stained in green, while several of them are stained in yellow; (**b**) G-A-A hydrogel: most of the cells are all stained in green; (**c**) F-A hydrogel: cells are all stained in green; (**d**) enlarged image of (**a**); (**e**) enlarged image of (**b**); (**f**) enlarged image of (**c**); (**g**) F-A-A hydrogel: most of the cells are alive and stained in green; (**h**) A hydrogel: a part of the cells are alive in green whereas some cells are dead in red; (**i**) A-A hydrogel: most cells are alive in green whereas several cells are dead in red; (**j**) enlarged image of (**g**); (**k**) enlarged image of (**h**), showing that some of the cells are dead in red; (**l**) enlarged image of (**i**), showing that some of the cells are dead in red.

After 10 days of in vitro 3D cultures, from the enlarged images of Figure 10a–c,g it can be seen clearly that the cell density in the hydrogels containing either gelatin or fibrinogen increases sharply (Figure 10d–f,j). Nearly all cells grow well and are stained in green inside the 3D hydrogels. However, when it comes to the control sets, A and A-A hydrogels, the increase in cell density is not obvious. In particular, in the A hydrogel, multiple cell deaths are detected in the field of view. Accordingly, the addition of gelatin and fibrinogen with suitable concentrations can significantly improve the cell compatibility of the pure alginate hydrogels.

## 3. Discussion

For in vitro 3D cell cultures, it is generally hoped that the hydrogels should have certain physicochemical properties with respect to their hardness, *WHC*, *SR* and swelling-erosion ratio in order to mimic the natural ECMs in organisms [26,27,28]. Several alginate-based hydrogels have been studied according to Table 1, and it was found that these hydrogels possess a good water permeability that can be used for in vitro cell 3D cultures. The Ca^2+^ crosslinked polyelectrolyte network of sodium alginate can provide “sacrificial bonds” to disperse the external stress and to provide rigid support for all of the above hydrogels. With the increase in the concentration of each polymer component, the *WHC* of hydrogels decreases, whereas the swelling ratio and hardness increase. This is because, with the increase in the polymer concentration, the space for water molecules is evidently reduced, so the micropore size resoundingly decreases. 

Gelatin, fibrinogen and agarose are three natural polymers with sufficient resources. When these polymers are added into the alginate solution and are crosslinked by using Ca^2+^, composite hydrogels are formed with some extra effects. For example, physically crosslinked agarose at 35–40 °C can form a stable interpenetrating network through hydrogen bonds with strong rigid structures, which is good for the 3D structural maintance [17,18,19]. For the alginate-based A-A hydrogel, the addition of agarose can obviously stabilize the water holding and swelling properties. A small amount (e.g., 0.5% *w*/*v*) of agarose is recommended to replace an equal proportion of alginate in the composite A-A hydrogel. Physically crosslinked gelatin at a temperature below 40 °C can also be used to increase the structural stability of the G-A hydrogel with a semi-solid gel state. The soft structures of the gelatin molecules can enlace the rigid alginate networks to provide a ductile connection between the two polymer chains, and to keep the hydrogel structure uniform and stable [29,30,31,32,33]. Fibrinogen without crosslinking can wrap the alginate networks and provide excellent biocompatibities. Additionally, for equal proportions of A and A-A hydrogels, the effect of fibrinogen on water retention is slightly higher than that of gelatin, whereas the effect of gelatin on the swelling rate is slightly higher than that of fibrinogen. Different hydrogel combinations and ratios can be selected and optimized to mimic different biomedical models according to clinical requirements [34,35,36]. 

The microstructure of the hydrogel determines the possibility of tissue/organ and tumor model formation. Since cells in the hydrogel survive and proliferate by adhering to the surface of the hydrogel network, the pore structure of the hydrogel should not be too large or too small, otherwise it will be difficult to communicate between cells, which will make it difficult to form the expected tissues/organs [37]. From the microstructure images, it can be seen that pure alginate hydrogels have an uneven pore structure after molding, so they are not suitable for use as biomolding materials alone (Figure 2). The addition of agarose, gelatin and fibrinogen can reduce the pore size of the alginate hydrogel and can provide more attachment sites for cells, leading to an increase in the cell’s survival rate and proliferation capability. A small amount of additions are preferable for cell survival inside the alginate-based hydrogels, since a large amount of additions could reduce the pore sizes and limit the cell proliferation. The flexible polymer chains provided by gelatin and fibrinogen enable the hydrogels to have a more uniform structural frame with a better cell compatibility [1,2,3,4,5,6]. 

The swelling-erosion ratio is another important property for hydrogels used for in vitro 3D cell cultures. Sometimes, it is expected that the polymer chains can be degraded at a certain ideal speed for new tissue and organ formation. Too fast a swelling-erosion ratio may cause the collapse of the hydrogel networks, whereas too slow a swelling-erosion ratio may restrict the growth of cells inside the hydrogels [20]. By comparing the swelling-erosion ratio of the alginate-based hydrogels, it is found that all of the swelling-erosion ratio curves rose briefly before falling steadily. The increase in the swelling-erosion ratio shows that the hydrogel’s weight is increased and accompanied by the expansion and softening of the hydrogels. This is because, when the hydrogel is immersed in PBS, the alginate molecules crosslinked by calcium ions can gradually dissolve in the medium and the crosslinking network can expand to some degree. Other components wrapped by the crosslinking network can continuously be lost into the medium, and a large amount of water can enter the hydrogel, resulting in the degradation curve increasing temporarily. With further degradation, the crosslinking network starts to break, which makes the water wrapped in the crosslinking network lose during the network breaking, thus forming the decline of the degradation curve in the later stage [38].

Agarose has a stable crosslinking network at room temperature. It is not easy to expand and break. Therefore, compared with the hydrogel system without agarose, the degradation curve of the whole system is gentler and eventually tends to be stable [39,40]. When the addition of agarose is very low, the components of agarose in the alginate-based hydrogels are insufficient and it is difficult to form a stable network under the condition of physical crosslinking. Therefore, the swelling–erosion ratio of delayed hydrogel is not obvious. However, when the addition of agarose is too high, the polymer network formed by agarose through hydrogen bonding can be firmly fixed to the alginate networks crosslinked by Ca^2+^, resulting in a slow or even no degradation of the hydrogels for a longer period (e.g., more than 12 days (Figure 5) [41,42,43].

By studying the growth states of cells on the 2D plates and in the 3D hydrogels, it is found that those cells grown on the 2D plates contact each other easily after several days of culture and inhibit the further increase in cell density [44,45,46,47,48]. However, in the 3D hydrogels, the contact inhibition phenomena can be effectively alleviated. There are many advantages for the cells to be cultured in the alginate-based 3D hydrogels [49,50,51,52]. Due to the fact that there are a large amount of pores and sites for cell attachment in the hydrogels, cells can divide and proliferate at 3Ds, resulting in contact inhibition delay [53,54,55,56]. With the degradation of some polymer networks, it will provide more space for cells to grow. Even if the contact inhibition phenomenon occurs in some areas, it may create some new growth space with the degradation of the networks, and thus cells may start to divide again. Especially, when 3D cultures of multiple types of cells, with a predesigned vascular network, reach a certain degree of density, a capillary network will be formed between the cells, so that the cells can fundamentally survive in vitro, and it is easier to meet the needs of transplantation of them into the organism.

## 4. Materials and Methods

### 4.1. Nature Polymer Component and Preparation of Hydrogels

Powder sodium alginate (Macklin, Shanghai, China), gelatin (Macklin, Shanghai, China, glue strength ~260 g), agarose (Biowest Agarose, Shanghai, China) and fibrinogen (BMASSAY, Beijing, China) were firstly irradiated in the UV for 60 min before being dissolved in deionized water and put into centrifugal tubes (Corning Incorporated, Corning, NY, USA). Then, 2% (*w*/*v*) calcium chloride (CaCl_2_, DAMAO, Tianjin, China) in deionized water was used to crosslink the alginate molecules in the polymer solutions. All operations were performed under sterile conditions.

For G-A and G-A-A hydrogels, gelatin, alginate or agarose was weighted, respectively, and deionized water was added to the mixture of two or three polymers to achieve the targeted concentration shown in Table 1. Then, the cocktail solution was heated in a water bath at 90 °C for 2 h to be completely dissolved. After dissolving, the cocktail solution was cooled below 40 °C and 2% (*w*/*v*) calcium chloride was used to crosslink for 30 min to form G-A or G-A-A hydrogels. The preparation of A and A-A hydrogels was the same as above. 

For F-A and F-A-A hydrogels, alginate and agarose were dissolved in deionized water at twice the concentration of Table 1 and heated in a water bath at 90 °C for 2 h to be completely dissolved. The fibrinogen powder was dissolved in aseptic phosphate buffer saline buffer (PBS) at twice the concentration of Table 1, placed on an aseptic shaker at 37 °C, where the shaking speed was adjusted appropriately, and shaken slowly for 2 h to completely dissolve. After that, the solution of alginate or agarose was cooled below 40 °C and mixed with fibrinogen at 1:1. Then, 2% (*w*/*v*) calcium chloride was used to crosslink for 30 min to form F-A or F-A-A hydrogels.

### 4.2. Water Holding Capacity Test

Three cylindrical samples with a diameter of 14 mm and a height of 9 mm were tested for *WHC* of the hydrogels. Briefly, the polymer solutions were firstly cooled at 4 °C for 30 min before being crosslinked for 30 min using CaCl_2_ solution. Then, the samples were washed with PBS 3 times (30 s each time) before being weighed using an electronic balance (ACCULAB, Sartorius Scientific Instruments, Beijing, China). The first weight after the surface moisture of the samples was fully removed was denoted as wet weight (*Ww*). The second weight after the samples were freeze-dried in a vacuum freeze-dryer (LGJ-10, Songyuan, Beijing, China) for 24 h was denoted as dry weight (*Wd*). The *WHC* was calculated according to Equation (1):

(1) $$WHC=\frac{Ww-Wd}{Wd}$$

### 4.3. Swelling Ratio Test

Three cylindrical samples with a diameter of 14 mm and a height of 9 mm were tested for *SR* of the hydrogels. Briefly, after cooling, crosslinking, cleaning and removing the surface moisture, the samples were dried in a constant temperature drying oven (JCZ-GPL, Jiacheng, Nantong, China) for 72 h (to ensure hydrogels dry thoroughly). The dry weight of the sample was measured and denoted as *Wd*. After the measurement, the samples were fully soaked in PBS and placed in a constant temperature drying oven at 37 °C for 48 h. The weight after water absorption was recorded as *Ww*. The *SR* was calculated according to Equation (2):

(2) $$SR=\frac{Ww-Wd}{Wd}$$

### 4.4. Swelling-Erosion Ratio

Three cylindrical samples with a 14 mm diameter and a 9 mm height were tested for swelling-erosion ratio of the hydrogels. Briefly, after cooling, crosslinking, cleaning and removing the surface moisture, the first weight of the samples was recorded as *W*0. The second weight, after the samples were soaked in PBS and incubated for 1, 2, 3, and 10 days, was recorded as *Wt*. The swelling-erosion ratio was calculated according to Equation (3):

(3) $$R=\frac{Wt-W0}{W0}\times 100\%$$

### 4.5. Mechanical Property Test

Three cylindrical samples with a 20 mm diameter and a 15 mm height were tested for hardness of the hydrogels. After cooling, crosslinking, cleaning and removing the surface water, the hardness was recorded using a Shore durometer (HT-6510OO, Lantai, Shenzhen, China). During the test, Shore durometer was pressed until it penetrated the hydrogel at a compression speed of 1 mm/s. The maximum number of the Shore durometer was recorded. This is also the yield limit of the hydrogels [53].

When the Shore durometer completely penetrated the hydrogel, the value at this time was hardness. When the hydrogel was compressed to less than 2/3 of the thickness of the original height, the value at this time was the elastic modulus, and the hydrogel could be observed to return to its initial state under the action of elastic deformation once loosening the Shore durometer.

### 4.6. Porous Structure of the Hydrogels Observation

After cooling, crosslinking, cleaning and removing the surface moisture, the hydrogel samples were frozen at −80 °C for 12 h and transferred to a vacuum freeze dryer for 24 h. 2 mm × 2 mm solid blocks of the dried samples were prepared before they were tightly fitted to the metal sample table with conductive adhesive. A cross section was sprayed with gold and observed using a field emission scanning electron microscope (JSM-7001F, JEOL, England, Beijing Dongfang Anuo Technology Co., Ltd., Beijing, China) [54].

### 4.7. Two-Dimensional Cell Cultures

Tongue carcinoma TCA 8113 cells were cultured in 37 °C, 5% CO_2_ incubator (BB 150, Thermo, Logan, UT, USA) using DMEM/HIGH glucose (HyClone, Logan, UT, USA) containing 10% fetal bovine serum culture medium. After counting the numbers, the cells were transferred to a 75 cm^2^ culture bottle (Corning Incorporated, Corning, NY, USA) with a density of 5 × 10^3^ cells/mL. The culture medium was changed every two days. Cell states were observed on day 1, 3, 5, 7, 10 and 13, respectively, under an optical microscope (CKX41, Olympus, Tokyo, Japan).

### 4.8. Cell Proliferation Test

Cell activity was detected using CCK-8 Cell Counting Kit (Vazyme, Nanjing, China) according to the instruction [55]. Briefly, after the TCA-8113 cells were added into the polymer solutions with a density of 5 × 10^3^ cells/mL, 100 µL of the cell-laden solution was moved to a well of a 96-well plate and crosslinked for 10 min using 20 µL of CaCl_2_ solution. Then, 200 µL of culture medium was added to each well for cell cultures. The culture medium was changed every day. After 1, 3, 5, 7 and 10 days of in vitro cultures, the culture medium in each well was removed before 100 µL culture medium and 20 µL CCK-8 solutions were added. After being cultured for 1 h in an incubator at 37 °C, the culture medium was transferred to a sterilized 96-well plate and its light absorption value, optical density (OD), was recorded using a microplate reader (Multiskan FC, Thermo, Scientific, Logan, UT, USA,) at a wavelength of 450 nm. The light absorption value of the cell-laden hydrogel was denoted as *ODt*, whereas the value of the control group was denoted as *ODn*. Cell proliferation rate was calculated via Equation (4):

(4) $$CV=\frac{ODt-ODn}{ODn}\times 100\%$$

### 4.9. Cell Activity Test in the Hydrogels

Acridine orange (AO)/propidium iodide (PI) double-dye kit (BestBio, Shanghai, China) was used for the live and dead identification of the cells according to the instructions [56]. Briefly, 1 mL of the cell-laden polymer solutions with a density of 2 × 10^6^ cells/mL was added into a 24-well plate and crosslinked for 10 min using 1 mL of CaCl_2_ solution. After crosslinking, 1 mL culture medium was added to each well for cell cultures. The culture medium was changed every 2 days. After 1, 3, 5, 7 and 10 days of in vitro cultures, a part of the cell-laden hydrogels was taken, replaced into a 5 mL EP tube and cleaned thoroughly with PBS before the pre-prepared AO/PI solution was added, incubated in dark at 4 °C for 20 min and observed under a laser confocal microscope (LSM, N1R, Nikon, Tokyo, Japan) at 488 nm exciting light.

### 4.10. Statistical Analysis

The results were presented as the mean ± standard deviation (SD). Statistical Product and Service Solutions (SPSS) 22.0 software (Chicago, IL, USA) was used for statistical analysis, and GraphPad Prism 8.0 software (San Diego, CA, USA) was used to plot. Single T test was used to compare the *WHC*, swelling rate, swelling-erosion ratio and hardness of various polymers. Cell growth rate was compared by single factor analysis of variance (ANOVA). A P-value less than 0.05 was considered statistically significant.

## 5. Conclusions

Through the research, we have obtained the hydrogels that are more suitable for tissue/organ molding or pathological model construction. G-A-A and F-A-A can both meet the needs of in vitro 3D cell cultures with excellent biocompatibilities. Ca^2+^ chemical crosslinking of algainte provides basic and enough physical and chemical properties for the alginate-based hydrogels with sufficient mechanical strengths and stable 3D structures. The addition of agarose as a complementary component on the one hand increased the number of pores of the hydrogels, appropriately reduced the pore structures and delayed the swelling-erosion ratios. On the other hand, it increased the hardness of the hydrogels and maintained the stability of the 3D structures of the hydrogels. The addition of gelatin and fibrinogen, on the one hand, makes the wall thickness of the hydrogel network uniform and stable. On the other hand, it builds a better external environment for cell survival. The optimized composite alginate-based hydrogels, with a proper microstructure, provide more space for the survival of cells, effectively increases the cell density, and greatly saves the cell culture cost with favorable conditions for cells to communicate inside and to finally form tissues/organs.

## Figures and Tables

**Table 1:** Composition and proportion of the alginate-based hydrogels.

Gelatin–alginate (G-A) Gelatin-alginate-agarose (G-A-A)	**Group**	**1**	**2**	**3**	**4**	**5**	**6**	**7**	**8**	**9**	**10**	**11**	**12**
	Gelatin (*w*/*v* %)	3.0	4.0	5.0	6.0	8.0	10.0	4.0	4.0	4.0	4.0	4.0	
	Alginate (*w*/*v*%)	1.5	1.5	1.5	1.5	1.5	1.5	0.5	1.0	2.0	2.5	3.0	
	Gelatin (*w*/*v* %)	3.0	4.0	5.0	6.0	8.0	10.0	4.0	4.0	4.0	4.0	4.0	4.0
	Alginate (*w*/*v*%)	1.0	1.0	1.0	1.0	1.0	1.0	0.5	1.5	2.0	1.0	1.0	1.0
	Agarose (*w*/*v*%)	0.5	0.5	0.5	0.5	0.5	0.5	0.5	0.5	0.5	0.2	0.8	1.0
Fibrinogen-alginate (F-A)													
	Fibrinogen (*w*/*v*%)	0.5	1.0	1.5	2.0	2.5	3.0	1.0	1.0	1.0	1.0	1.0	
	Alginate (*w*/*v*%)	1.0	1.0	1.0	1.0	1.0	1.0	0.5	1.5	2.0	2.5	3.0	
Fibrinogen-alginate-agarose (F-A-A)													
	Fibrinogen (*w*/*v*%)	0.5	1.0	1.5	2.0	2.5	3.0	1.0	1.0	1.0	1.0	1.0	1.0
	Alginate (*w*/*v*%)	0.5	0.5	0.5	0.5	0.5	0.5	0.2	0.8	1.0	0.5	0.5	0.5
	Agarose (*w*/*v*%)	0.5	0.5	0.5	0.5	0.5	0.5	0.5	0.5	0.5	0.2	0.8	1.0
Alginate (A)													
	Alginate (*w*/*v*%)	0.5	1.0	1.5	2.0	2.5	3.0						
Alginate-agarose (A-A)													
	Alginate (*w*/*v*%)	1.0	1.0	1.0	1.0	1.0	1.0	0.5	1.5	2.0	0.5	0.5	0.5
	Agarose (*w*/*v*%)	0.2	0.5	0.8	1.0	1.5	2.0	0.5	0.5	0.5	0.2	0.8	1.0

**Table 2:** Optimum ratio of polymer components in each group.

	G-A	A	G-A-A	A-A	F-A	A	F-A-A	A-A
Gelatin (*w*/*v*%)	4		4					
Fibrinogen (*w*/*v*%)					1		1	
Alginate (*w*/*v*%)	1.5	1.5	1	1	1	1	0.5	0.5
Agarose (*w*/*v* %)			0.5	0.5			0.5	0.5

## Data Availability

All the data are reliable, and anyone can email the author to request the original data.
